# A test of male infanticide as a reproductive tactic in a cichlid fish

**DOI:** 10.1098/rsos.160891

**Published:** 2017-03-08

**Authors:** Shagun Jindal, Aneesh P. H. Bose, Constance M. O'Connor, Sigal Balshine

**Affiliations:** Aquatic Behavioural Ecology Laboratory, Department of Psychology, Neuroscience, and Behaviour, McMaster University, 1280 Main Street West, Hamilton, OntarioCanadaL8S 4K1

**Keywords:** cannibalism, parental care, takeovers, group living, Lake Tanganyika, cooperative breeding

## Abstract

Infanticide and offspring cannibalism are taxonomically widespread phenomena. In some group-living species, a new dominant individual taking over a group can benefit from infanticide if doing so induces potential mates to become reproductively available sooner. Despite widespread observations of infanticide (i.e. egg cannibalism) among fishes, no study has investigated whether egg cannibalism occurs in fishes as a result of group takeovers, or how this type of cannibalism might be adaptive. Using the cooperatively breeding cichlid, *Neolamprologus pulcher*, we tested whether new unrelated males entering the dominant position in a social group were more likely to cannibalize eggs, and whether such cannibalism would shorten the interval until the female's next spawning. Females spawned again sooner if their broods were removed than if they were cared for. Egg cannibalism occurred frequently after a group takeover event, and was rarer if the original male remained with the group. While dominant breeder females were initially highly aggressive towards newcomer males that took over the group, the degree of resistance depended on relative body size differences between the new pair and, ultimately, female aggression did not prevent egg cannibalism. Egg cannibalism, however, did not shorten the duration until subsequent spawning, or increase fecundity during subsequent breeding in our laboratory setting. Our results show that infanticide as mediated through group takeovers is a taxonomically widespread behaviour.

## 1. Background

One of the most dramatic examples of sexual conflict in the animal kingdom is infanticide perpetrated by newly dominant male lions, *Panthera leo*. When male lions take over a pride of females, they will kill cubs and the termination of maternal provisioning allows females to mate again sooner [1–3]. Infanticide defined as the direct killing of, or lethal curtailment of investment into, conspecific young [4] therefore grants ‘takeover’ males sexual access to females, increasing the males' reproductive success, albeit at a cost to the females [3,5]. The sexual selection hypothesis for infanticide predicts that offspring will be killed when: (i) an individual in a dominant breeding position is replaced by a new, unrelated, individual; (ii) infanticide elicits a state of receptivity in the potential mates of the perpetrator who have had their care terminated; and (iii) subsequent reproduction between the perpetrator and the mates is enhanced either through shortened interbirth/interhatch intervals, increased fecundity, or both. The costs to the original parents are also thought to have driven the evolution of numerous counter-strategies, including female aggression against usurping males [3,6,7].

The sexual selection hypothesis for infanticide was originally formulated to describe the behaviour of Hanuman langurs, *Semnopithecus entellus* [5], and the hypothesis has received support from a number of mammalian studies [7,8]. Mammals are particularly apt for illustrating the adaptive benefits of infanticide, because the costs associated with female lactation are so prohibitive that they generally preclude the raising of multiple broods simultaneously, and these costs can be avoided by eliminating dependent offspring [6,9]. The sexual selection hypothesis has received far less research attention in non-mammalian species ([3]; but see also [10]) and therefore garnered only limited empirical support across taxa. This is despite widespread understanding that parental care imposes reproductive costs to caregiving individuals [11,12]. Although parental care has been shown to reduce female breeding frequency by increasing interspawn intervals in numerous fish species (*Oreochromis mossambicus*, [13]; *Haplochromis argens*, [14]; *Sarotherodon galilaeus,* [15]), no study has yet tested the sexual selection hypothesis for infanticide in fishes.

Here, we used the cooperatively breeding, group-living cichlid fish, *Neolamprologus pulcher*, to conduct the first test of the sexual selection hypothesis for infanticide in fishes. In fishes, infanticide is always committed through cannibalism [16] and so we henceforth refer to infanticide as egg cannibalism in this study. We first examined whether the cessation of parental care would shorten interspawn intervals and/or enlarge subsequent broods by conducting brood removals in the early stages of parental care. We next staged scenarios in which breeding groups were taken over by a new, unrelated dominant male to test whether egg cannibalism subsequently occurred. We then tested whether female aggression towards newcomer males was effective in preventing egg cannibalism, and whether egg cannibalism provided the takeover male with a reproductive benefit. We predicted that: (i) social groups of *N. pulcher* would spawn again sooner when their broods were removed, thereby ending parental care early, compared to when their broods were cared for; (ii) new takeover males would cannibalize any eggs currently receiving care from the social group; (iii) maternal aggression would counter the takeover male's cannibalistic efforts, particularly when the female was well matched in terms of body size to the takeover male; and (iv) egg cannibalism would benefit the takeover male by shortening the interval until subsequent spawning and/or by increasing female fecundity in the following breeding event.

## 2. Methods

### 2.1. Study species

Social groups of *N. pulcher* in the wild consist of a dominant breeding male and female and 0–20 subordinate helpers ordered in a size-based dominance hierarchy [17]. Average group size in the wild varies between 7 [18] to 9 [19]. While both breeders and helpers maintain the territory, defend it from competitors and predators, and care for offspring, the breeder female tends to be the most active in all these respects [18,20]. Males typically disperse further than females and have shorter tenure in the dominant position than do females, resulting in males generally having fewer reproductive opportunities over their lifetimes than females [21]. From time to time, a new breeding male from outside of the social group (often a neighbour) will attempt to take over a recently vacated breeding position (often vacant due to predation) or to challenge the current breeding male for their position [21,22]. Like many cichlids, *N. pulcher* breed regularly throughout the year and groups tend to produce eggs once per month [23,24].

### 2.2. Does egg removal expedite subsequent reproduction?

Between April 2011 and September 2012, 10 social groups of *N. pulcher* were held in the laboratory at McMaster University, Hamilton, Canada in 189-litre aquaria. The aquaria were maintained at 25 ± 2°C with a 13 : 11 h light–dark cycle and contained crushed coral substrate. Housing conditions were environmentally controlled and kept stable across the entirety of the observation period. Each tank was also given two halved flowerpots, which are used for shelter and breeding. Each flowerpot provided ample surface area for egg attachment ensuring that spawning was never space-limited. Fish were fed Nutrafin® cichlid food flakes daily and the groups were breeding consistently in the laboratory (i.e. at least once every two months). For each of these groups, two broods were haphazardly chosen for complete removal immediately upon their discovery, and another two broods were allowed to be raised by their social group. We recorded the dates of subsequent spawning in order to calculate interspawn intervals (*N* = 40 interspawn intervals calculated, *N* = 10 groups). When the broods were clearly visible, we also recorded brood size (*N* = 26 brood sizes recorded).

All statistical analyses were performed in R (v. 3.2.3) [25]. To assess whether brood removal influenced a social group's interspawn interval or brood size, we fit two linear mixed-effects models (LMMs), including either interspawn interval (in days) or brood size (egg number) as the response variable, treatment (removed versus not removed) as a fixed effect and GroupID as a random intercept (data provided in electronic supplementary material).

### 2.3. Does group takeover incite egg cannibalism and expedite subsequent reproduction?

We conducted this experiment between April 2015 and March 2016 at McMaster University using 39 social groups of *N. pulcher* held in 189-litre aquaria. Each social group consisted of a dominant male and female breeding pair plus three or four subordinate helpers. Aquarium conditions were environmentally controlled and kept stable as described above, but in this experiment each social group was fed a precise diet of 75 mg of Nutrafin® cichlid food flakes daily.

Here, we simulated the natural situation often observed in the wild in which a neighbouring male takes over a social group. Under natural conditions, it is common for the dominant male breeding position to be filled by a closely neighbouring male, who either actively competes for the position by ousting the original male through a takeover or waits for the position to become vacant through predation [22,26]. For example, Stiver *et al*. [22] showed in the wild that 71% of experimentally vacated male breeder positions were filled by neighbouring males taking over the group. We therefore held a neighbouring adult male in an end-compartment of the same aquarium as our social groups, where he was visible to the social group but separated by a removable clear barrier. Each social group was inspected daily for eggs. When a brood of eggs was detected, it was photographed, and the group was haphazardly assigned to either the takeover (*N* = 25) or control (*N* = 14) condition. In takeover groups, the dominant breeding male was removed. The group was permitted 15 min to recover from this disturbance before the barrier was removed, thereby allowing the neighbouring male to ‘take over’ the social group. In control groups, the dominant male was removed, but after 15 min, he was returned to his social group while the neighbouring male and barrier were removed. We video-recorded all behaviours for 1 h, and then photographed the broods after this hour and again after 24 h. We quantified brood size from the digital photos using ImageJ (v 1.48), and used these numbers to calculate the proportion of each brood that survived to the 1 h and 24 h time points. We inspected all groups daily for the next two months and recorded any subsequent spawning events.

From the recorded videos, a single observer scored the first 20 min of interactions between the original breeding pair in control groups, and between the dominant female and the new male in takeover groups. All aggressive and submissive behaviours performed and received were scored [27], and used to calculate a resistance index for each female as a measure of her ability to dominate the male: Resistance Index = (Aggression_given_ + Submission_received_) − (Aggression_received_ + Submission_given_). Interactions with helpers were rare and therefore were not analysed. The observer also recorded whether or not egg cannibalism occurred, the identities of all egg cannibals and how many eggs were cannibalized over the entirety of the 60 min trial.

Prior to the manipulation, group size did not differ between takeover and control groups, nor did brood sizes, nor the body sizes of dominant females, original dominant males and isolated/takeover males (all *p* > 0.22). We fit a linear model (LM) to test how female resistance differed between the takeover and control groups and how it correlated with brood size and male–female size disparity (% difference in standard length). Next, we tested whether groups differed in terms of the proportions of the brood surviving to the 1 h and the 24 h time points. For each time point, we fit a generalized linear model, specifying a quasi-binomial error distribution (GLMqb) suitable for overdispersed data and proportion data [28]. We specified treatment group, initial brood size and female resistance index as independent variables. All two-way interactions were tested and removed when non-significant. We then employed a hurdle model to test how takeovers affected subsequent reproduction. We fit a binary logistic regression to compare groups for their likelihood of having a subsequent spawning within the two-month post-manipulation monitoring period. We then focused only on trials in which a subsequent spawning occurred (*N* = 33), and fit two linear models to see how takeovers affected the number of days until the next spawning (log-transformed), and if takeovers affected subsequent brood size. For both these models, treatment group was specified as the independent variable (data provided in electronic supplementary material).

## 3. Results

### 3.1. Removing broods shortens interspawn intervals

**Figure 1.**
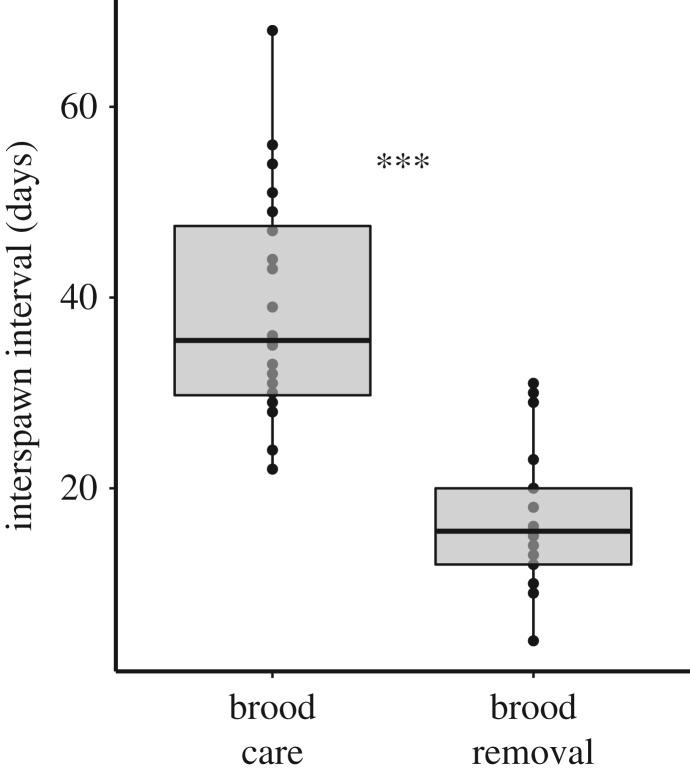
Interspawn intervals shorten when broods are removed. *** indicates *p* < 0.0001.

In 2011 and 2012, the 10 *N. pulcher* social groups studied spawned on average every 28.0 ± 14.9 days (mean ± s.d.). Groups that had their broods removed re-mated sooner (every 16.9 ± 7.2 days) compared with groups that cared for their broods (every 39.0 ± 12.1 days; LMM, *t* = 7.7, *p* < 0.0001, marginal *R*^2^ = 0.55, conditional *R*^2^ = 0.64; figure 1). However, subsequent brood sizes were not influenced by whether the previous brood was removed (84.1 ± 65.8 eggs) or cared for (49.8 ± 26.5 eggs; LMM, *t* = −1.6, *p* = 0.13, marginal *R*^2^ = 0.09, conditional *R*^2^ = 0.18).

### 3.2. Group takeovers are associated with female aggression and egg cannibalism

**Figure 2.**
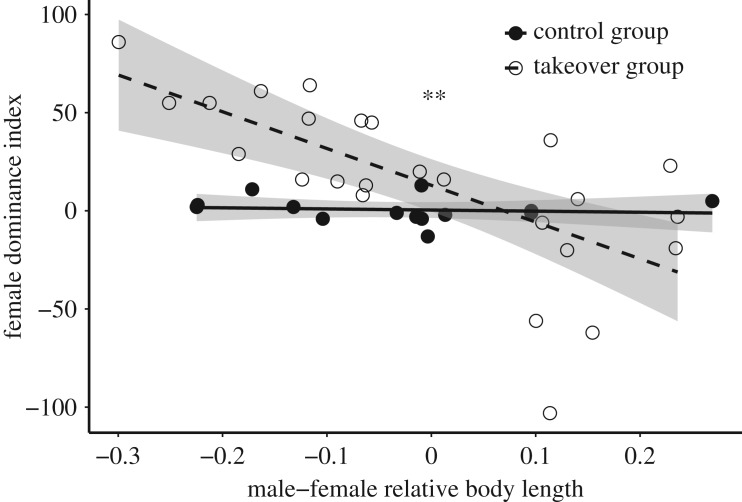
Female resistance to male takeover was high when females were larger than the takeover males, but diminished when the takeover males were larger (dashed line). This pattern was not observed in the control groups (solid line). 95% confidence intervals shown. ** indicates *p* < 0.01.

More aggression was observed in the takeover groups (mean ± s.d., 67.1 ± 42.4 aggressive acts/20 min) than in control groups (13.3 ± 12.0 aggressive acts/20 min). While female resistance was not related to brood size (LM, *t* = −0.16, *N* = 39, *p* = 0.88), it was correlated with male–female size disparity, but only in the takeover groups (LM, interaction, *t* = −2.80, *N* = 39, *p* = 0.008, adjusted *R*^2^ = 0.42; figure 2).

**Figure 3.**
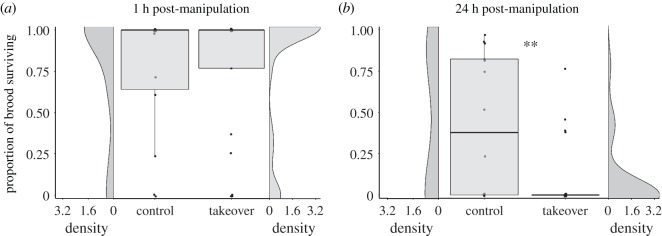
Proportion of brood surviving at (*a*) 1 h and (*b*) 24 h post-manipulation. Kernel density plots are also presented to better visualize the shape of the skewed data in each boxplot. ** indicates *p* < 0.01.

Over the 1 h trials, egg cannibalism was directly observed in *both* control and takeover groups. Over the first hour of the trials, group takeovers resulted in the cannibalism of 177 eggs (in total), while controls resulted in the cannibalism of 58 eggs (in total). Cannibalism was directly observed in seven of the 25 takeover trials (all committed by the takeover male) and in five of the 14 control groups (four of these were committed by the control breeder male, and once by the control breeder female). After 1 h, there were no differences between the control and takeover groups in terms of the proportions of the broods surviving (GLMqb, *t* = −0.84, *N* = 39, *p* = 0.41; figure 3*a*), though larger broods were more likely to have a higher proportion of offspring surviving after 1 h (GLMqb, *t* = 2.05, *N* = 39, *p* = 0.048). After 24 h, however, far fewer offspring remained in the takeover groups than the control groups (GLMqb, *t* = −2.77, *N* = 39, *p* = 0.009; figure 3*b*). Female resistance was not related to the proportion of offspring surviving at either time point (both *p* > 0.2).

### 3.3. Group takeovers and egg cannibalism are not associated with expedited reproduction

Thirty-three of the 39 social groups used in our experiment spawned again within two months. Takeover events did not increase the likelihood of a second spawning occurring in this timespan (binary logistic regression, *z* = −1.02, *N* = 39, *p* = 0.31), or reduce the days until the second spawning (LM, *t* = 0.22, *N* = 33, *p* = 0.82, multiple *R*^2^ = 0.002). The mean (±s.d.) interspawn interval was 24.2 ± 13.6 days for control groups and 24.2 ± 13.0 days for takeover groups. The subsequent brood size also did not differ between treatments (62.7 ± 20.1 eggs for control groups and 52.2 ± 27.7 eggs for takeover groups; LM, *t* = −1.18, *N* = 33, *p* = 0.25, multiple *R*^2^ = 0.04).

## 4. Discussion

Infanticide and cannibalism following group takeovers have been documented in numerous mammalian and avian species that display parental care [5–8]. However, to the best of our knowledge this phenomenon had not yet been studied in any fish species. With hierarchical group living and offspring care, *N. pulcher* provides an ideal model to investigate this phenomenon. Furthermore, though egg cannibalism is widespread among fishes [29–31], it has not been well studied in *N. pulcher*, and never in the context of group takeovers.

Group takeovers were associated with high levels of egg cannibalism. Egg survival after 24 h was much lower in the takeover condition compared to the control condition. Thus, it appears that takeover males benefit more by cannibalizing eggs immediately rather than allowing those offspring to augment the group in the future as helpers (e.g. [19,32]). Intriguingly, we showed that removing broods could shorten interspawn intervals in *N. pulcher*, illustrating that cannibalistic takeover males could *potentially* benefit in this respect. However, in our takeover experiment we found no evidence that takeovers and egg cannibalism in *N. pulcher* sped up future reproduction or led to larger subsequent broods. We speculate that the abundant risk-free food provided in our laboratory may have influenced this result. Throughout the study, all groups were fed a standardized diet that was probably generous in comparison to what these fish manage to acquire in the wild [33]. Stronger resource limitation or riskier foraging situations, such as those experienced naturally in the wild, may lead to more pronounced trade-offs between current parental care and egg production. In support of this idea, breeding in the wild occurs every one to two lunar cycles [33], while breeding in the laboratory can occur twice per month, suggesting that egg production is resource-limited. A critical future test will be to combine group takeovers in the wild with a manipulation of the takeover male's ability to cannibalize eggs.

We were surprised to also observe filial egg cannibalism occurring in control trials. Some egg cannibalism does occur naturally in this cooperative breeding fish [34]. Previous reports of egg cannibalism in *N. pulcher* have been in relation to dominant females cannibalizing the non-kin broods of subordinate females as a means of suppressing subordinate reproduction [35]. However, egg cannibalism in this species has never been directly observed in the wild. Therefore, we cannot rule out that filial cannibalism is not a natural component of *N. pulcher* parental care. Furthermore, our handling disturbance may also have induced some filial cannibalism in our control groups, and indeed also in our takeover groups.

Females were aggressive towards takeover males, yet they did not ultimately prevent cannibalism; the majority of takeovers resulted in complete brood mortality. In many species, maternal aggression has been hypothesized to be a female counter-tactic to male infanticide. However, support for this idea is mixed, as maternal aggression often appears to delay, rather than prevent, infanticide [3,7]. If maternal aggression in *N. pulcher* were indeed a tactic meant to prevent egg cannibalism, then we predicted female aggression towards the takeover male to be positively related to brood size. However, we did not detect this relationship in our data. Aggression may instead be a way to establish dominance and familiarity between the new pair of fish. The use of aggression in establishing dominance hierarchies has been well documented in this species [33]. Alternatively, it is possible that female aggression is still an effective cannibalism-prevention tactic in the wild. In our laboratory conditions, takeover males did not have the opportunity to retreat away from a contest (confined within the aquaria), and so females may not have been able to truly expel the new male from the territory.

In this study, we staged group takeovers in *N. pulcher* to assess the role of egg cannibalism and infanticide as an adaptive reproductive tactic. Conventional parental investment theory predicts that infanticide would occur because the putative costs of killing unrelated young are low. Here, we tested an alternative, but non-mutually exclusive explanation, that the benefits can also be high. While takeovers were associated with high offspring mortality, we were unable to show that egg cannibalism shortens interspawn intervals or increases subsequent brood sizes. We urge that future work be undertaken in the field to further test this hypothesis under more naturalistic conditions. Our study demonstrates that infanticide following group takeovers extends to fishes and suggests interesting new avenues for research on the adaptive benefits of infanticide.

## Supplementary Material

Jindal et al. trial data

## Supplementary Material

Jindal et al. brood removal

## Data accessibility

Data deposited at Dryad. http://dx.doi.org/10.5061/dryad.gm187 [36].
